# Effect of raloxifene on IGF-I and IGFBP-3 in postmenopausal women with breast cancer

**DOI:** 10.1054/bjoc.2001.2191

**Published:** 2001-12

**Authors:** R Torrisi, L Baglietto, H Johansson, G Veronesi, B Bonanni, A Guerrieri-Gonzaga, B Ballardini, A Decensi

**Affiliations:** Division of Chemoprevention, Laboratory Medicine Unit, Division of Epidemiology and Biostatistics and Senology, European Institute of Oncology, via Ripamonti 435, Milan, 20141, Italy

**Keywords:** breast cancer prevention, SERMs, IGF system

## Abstract

The effect on the IGF system of 60 mg and 600 mg daily of raloxifene administered for 2 weeks prior to surgery was investigated in 37 postmenopausal women with breast cancer. Raloxifene significantly decreased insulin-like growth factor (IGF-I) as compared to placebo (*P* < 0.05) with no dose–response relationship. No significant change was observed in IGFBP-3, while the IGF-I/IGFBP-3 molar ratio was decreased by treatment, with a statistically significant effect only for the higher dose. Given that high plasma levels of IGF-I have been suggested as a risk factor for breast cancer, these findings provide further support for the potential activity of raloxifene in breast cancer prevention. © 2001 Cancer Research Campaign http://www.bjcancer.com


*British Journal of Cancer* (2001) **85**(12), 1838–1841
© 2001 Cancer Research Campaign
doi: 10.1054/ bjoc.2001.2191, available online at http://www.idealibrary.com on IDEAL

http://www.bjcancer.com

### Effect of raloxifene on IGF-I and IGFBP-3 in postmenopausal women with breast cancer

**R Torrisi[1], L Baglietto[3], H Johansson[1,2], G Veronesi[4], B Bonanni[1], A Guerrieri-Gonzaga[1], B Ballardini[4] and A Decensi[1]**

[1]Division of Chemoprevention; [2]Laboratory Medicine Unit; [3]Division of Epidemiology and Biostatistics and [4]Senology, European Institute of Oncology, via Ripamonti 435, 20141 Milan, Italy

**Summary** The effect on the IGF system of 60 mg and 600 mg daily of raloxifene administered for 2 weeks prior to surgery was investigated in 37 postmenopausal women with breast cancer. Raloxifene significantly decreased insulin-like growth factor (IGF-I) as compared to placebo ($P < 0.05$) with no dose–response relationship. No significant change was observed in IGFBP-3, while the IGF-I/IGFBP-3 molar ratio was decreased by treatment, with a statistically significant effect only for the higher dose. Given that high plasma levels of IGF-I have been suggested as a risk factor for breast cancer, these findings provide further support for the potential activity of raloxifene in breast cancer prevention. © 2001 Cancer Research Campaign http://www.bjcancer.com

**Keywords**: breast cancer prevention; SERMs; IGF system

The IGF system is currently recognized as a risk factor for the major epithelial cancers (Burroughs et al, 1999). Circulating levels of IGF-I derive primarily from hepatic synthesis and whether they affect IGF-I tissue bioactivity is not well known (Pollak, 2000). However, the positive association between high plasma IGF-I levels and cancer risk suggest that not only does it act as a tissue growth factor, but circulating IGF-I also exerts an endocrine role (Pollak, 1998). This evidence lends further credence to the hypothesis that strategies designed to lower circulating levels of IGF-I may be relevant in interfering with cancer initiation and/or progression.

In particular, a large amount of evidence is accumulating concerning the role played by IGF-I and its major binding protein IGFBP-3 in predicting pre-menopausal breast cancer risk (Hankinson et al, 1998) and prognosis (Vadgama et al, 1999). Both hormonal and non-hormonal agents, namely tamoxifen and the synthetic retinoid fenretinide, have been shown to lower circulating IGF-I (Pollak, 1998; Torrisi et al, 1993) and to decrease breast cancer incidence (Fisher et al, 1998; Veronesi et al, 1999), although the relationship between these two effects is currently under investigation.

The benzothiophene selective oestrogen receptor modulator (SERM) raloxifene was approved by the FDA for use in the prevention and treatment of osteoporosis in postmenopausal women. Within a randomized multicentric trial carried out in postmenopausal osteoporotic women using risk of vertebral fractures as the main endpoint (MORE trial), a 76% reduction in invasive breast cancer was observed after treatment with raloxifene 60 mg or 120 mg for 40 months (Cummings et al, 1999). Although experience with raloxifene in advanced disease is limited (Buzdar et al, 1988; Gradishar et al, 2000), it has been shown that raloxifene is able to decrease a marker of breast tumour proliferation (Ki67) when administered prior to surgery in women with postmenopausal breast cancer (Dowsett et al, 2001). In the present study, we investigated the effects of short-term administration of raloxifene in two different doses, and the occurrence of a dose–response relationship on IGF-I, IGFBP-3 and the IGF-I/IGFBP-3 ratio levels in postmenopausal women with primary breast cancer.

*Received 21 February 2001*
*Revised 29 August 2001*
*Accepted 3 October 2001*

*Correspondence to:* A Decensi

### MATERIALS AND METHODS

### Study design and subjects

A randomized, phase II placebo-controlled multicentric trial was designed to assess the activity of two different doses of raloxifene (60 mg/d vs 600 mg/d) for 14 d before definitive surgery in postmenopausal breast cancer patients, as described previously (Dowsett et al, 2001). Women aged 50–80, with newly diagnosed stage I–II breast cancer were eligible. Patients gave their written informed consent. A total of 167 women were enrolled in 10 centres in the UK and at the European Institute of Oncology in Italy. The study was approved by the Ethical Review Board at each centre.

The present study on the IGF system was conducted in a subgroup of 37 women admitted to the European Institute of Oncology of Milan who were enrolled in this multicentric trial.

Blood samples were obtained at randomization and after a 14-day treatment course. Plasma and serum were separated by centrifugation and aliquots were stored at –20˚C until assayed. All assays were performed by one of the authors of this study (HJ), who was blinded as to treatment allocation.

### Assay methods

Plasma concentrations of IGF-I were determined by enzyme-linked immunosorbent assay (ELISA) kits purchased from Diagnostic Systems Laboratories, Inc. (Webster, TX, USA). The sensitivity of the assay was 0.03 ng/ml; intra- and interassay coefficients of variation were 4.9% and 7.9%, respectively. In order to avoid interference by IGF-binding proteins, the assay method was preceded by an acid/ethanol extraction procedure, as previously described (Daughaday et al, 1980).

Serum IGFBP-3 concentrations were measured by ELISA kits purchased from Diagnostic Systems Laboratories, Inc. (Webster, TX, USA). The sensitivity of the assay was 0.04 ng/ml; intra- and interassay coefficients of variation were 3.3% and 5.7%, respectively.

### Statistical methods

The concentrations of IGF-I, IGFBP-3 and the IGF-I/IGFBP-3 ratio measured before and after the treatment were analysed as repeated measures data. The percentage change, calculated as the ratio between the difference of post- and pre-treatment values and pre-treatment values, was assessed for each response variable. Since age and body mass index (BMI) may affect IGF-I levels, they were included in the model as categorized variables. In order to take into account the correlation between repeated measures of the response variables within the same subject, data were analyzed using a mixed model with the intercept fitted as a random effect at subject level (Goldstein, 1995). Analysis was performed using log-transformed data. Data were analysed using the SPLUS 2000 (MathSoft Inc., Seattle, WA, USA). Statistical significance was defined as two-tailed $P < 0.05$.

### RESULTS

The main patient characteristics are described in Table 1. Age and BMI, which both may affect the IGF system, were comparable in the groups. At baseline, no significant difference in IGF-I, IGFBP-3 or their molar ratio was observed between the three groups. As for the expression of oestrogen receptors, there was a lower proportion of subjects with negative receptors in the group of women treated with raloxifene as compared with placebo. Results of the IGF changes after treatment are summarized in Table 2.

**Table 1** Patient characteristics at the baseline

| | Placebo *n* = 11 | RAL 60 *n* = 13 | RAL 600 *n* = 13 |
|---|---|---|---|
| Age (yrs) | 63 ± 7 | 60 ± 7 | 62 ± 6 |
| BMI (kg/m$^2$) | 25.2 ± 5.6 | 26.6 ± 5.1 | 26.2 ± 4.7 |
| ER status (+/–/uk) | 6/5/0 | 9/2/2 | 9/2/2 |
| IGF-I (nM/L) | 24.2 ± 10.9 | 20.8 ± 6.1 | 22.7 ± 6.1 |
| IGFBP3 (nM/L) | 171.9 ± 28.9 | 167.7 ± 19.3 | 168.1 ± 31.3 |
| IGF ratio | 0.14 ± 0.04 | 0.12 ± 0.03 | 0.13 ± 0.02 |

RAL 60 = raloxifene 60 mg/day; RAL 600 = raloxifene 600 mg/day; BMI = body mass index; ER = estrogen receptor; +/–/uk = positive/negative/unknown; IGF ratio = IGF-I/IGFBP-3 molar ratio. Data are expressed as mean ± SD value.

**Table 2** Percentage change from baseline after 14 days of treatment

| | Placebo (*n* = 11) | RAL 60 (*n* = 13) | RAL 600 (*n* = 13) |
|---|---|---|---|
| IGF-I | – 8 ± 5 | – 21 ± 4[a] | – 23 ± 4[a] |
| IGFBP-3 | – 5 ± 3 | – 10 ± 3 | – 1 ± 3 |
| IGF ratio | – 3 ± 5 | – 13 ± 4 | – 23 ± 3[b,c] |

RAL 60 = raloxifene 60 mg/day; RAL 600 = raloxifene 600 mg/die; IGF ratio = IGF-I/IGFBP-3 molar ratio. [a]$P < 0.05$ vs placebo; [b]$P < 0.001$ vs placebo; [c]$P < 0.05$ vs RAL 60. Data are expressed as mean ( ± SE) value.

After 2 weeks of treatment, IGF-I declined significantly in the raloxifene (RAL) 60 and the RAL 600 groups as compared with placebo ($P < 0.05$) (Figure 1). The magnitude of IGF-I decline was not significantly different between the two doses (Figure 1). Comparable results were obtained when the analysis was performed while adjusting for IGFBP-3 (data not shown).

IGFBP-3 was significantly decreased by treatment as compared to baseline in the RAL 60 group with a mean percentage change of –10% ± 3. However, when a comparison among groups was performed, this decrease was not significantly different from the 5% reduction observed in the placebo group and from the absence of change observed in the RAL 600 group (Figure 1). No significant difference among groups was also observed when the analysis was performed while adjusting for IGF-I (data not shown).

As a consequence of these results, the IGF-I/IGFBP-3 molar ratio was significantly decreased by treatment and a trend towards a dose–response effect was evident, with a mean percentage change of –13% ± 3 and –23% ± 3 in the RAL 60 and RAL 600 groups, respectively (Figure 1).

Similar results were observed when the effect of raloxifene on the IGF system was investigated separately according to the hormone receptor status (data not shown). Although the number of ER-negative tumours was too small to draw any conclusions on the ability of the ER status to affect raloxifene activity, this analysis was aimed at confirming the consistency of the main effect in subgroups.

Age and BMI did not modify the effect of treatment on IGFs (data not shown).

### DISCUSSION

The use of intermediate endpoint biomarkers for a rapid evaluation of the activity of preventive drugs is strongly encouraged in chemoprevention trials, in the attempt to reduce the number of subjects and the length of observation required (Kelloff et al, 1994). Recent epidemiological studies show circulating IGF-I to be a candidate endpoint biomarker of carcinogenesis in several epithelial tissues (Burroughs et al, 1999). While the efficacy of raloxifene use in breast cancer prevention is currently undergoing evaluation in comparison with tamoxifen in a randomized trial (STAR trial) in the USA, the short-term effects of the drug on proliferation and circulating IGF-I levels have been investigated in an attempt to further characterize a biological mechanism for a possible breast cancer prevention effect.

The results of the present study show that raloxifene decreases circulating IGF-I and the IGF-I/IGFBP-3 molar ratio in women with postmenopausal breast cancer. The magnitude of this decrease is comparable to that observed with other SERMs such as tamoxifen (Lonning et al, 1992) and droloxifene (Helle et al, 1996), although raloxifene was administered for a shorter period (14 days vs 1–3 months for other SERMs). Just as with tamoxifen (Decensi et al, 1998), but in contrast with droloxifene (Helle et al, 1996), no dose–response effect was observed on IGF-I decline. A trend towards a greater effect on the IGF-I/IGFBP-3 ratio was evident for the higher dose, probably due to the IGFBP-3 decline in the lower dose group. The latter finding should be regarded with some caution, since we cannot rule out the possibility that it occurred by chance.

The clinical implication of the present findings is still unresolved. Circulating IGF-I has not been found to be related to breast cancer risk in postmenopausal women in either of the two large

© 2001 Cancer Research Campaign

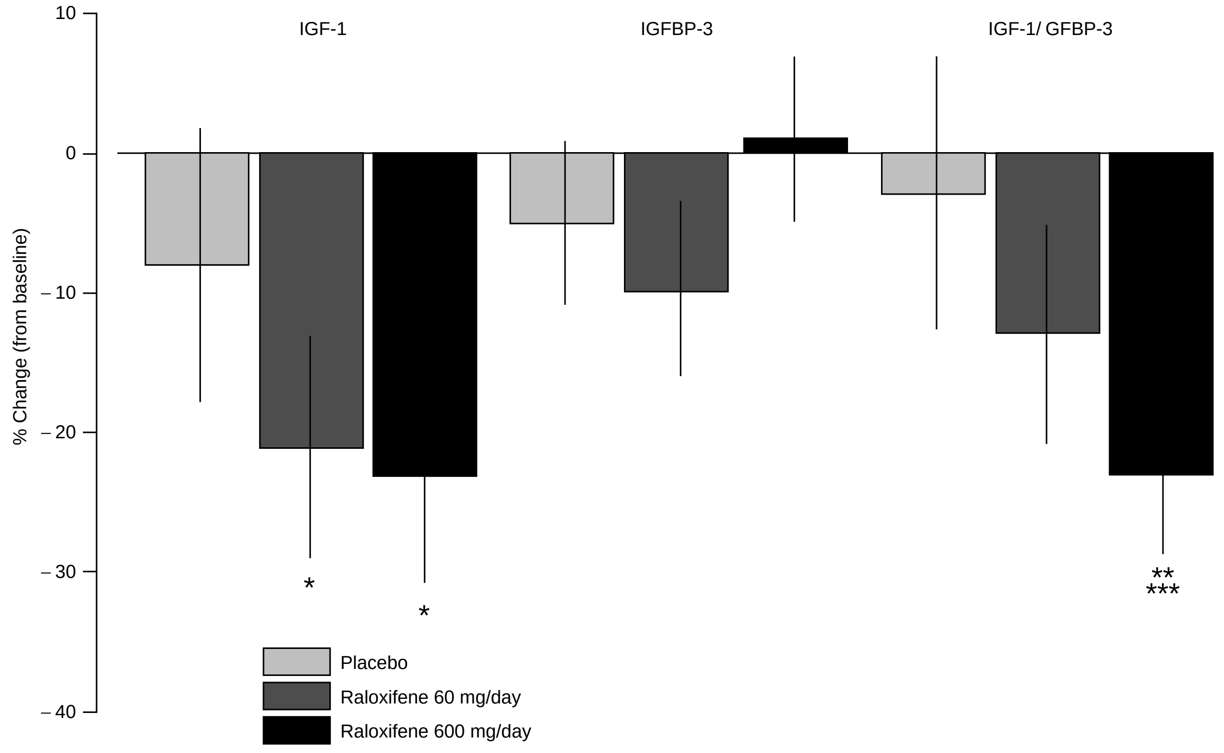

**Figure 1** Percentage (%) change ± 95% CI of IGF-I, IGFBP-3 and the IGF-I/IGFBP-3 ratio after 2 weeks of treatment. * $P < 0.05$ vs placebo; ** $P < 0.001$ vs placebo; *** $P < 0.05$ vs RAL 60

prospective studies (Hankinson et al, 1998; Toniolo et al, 2000); therefore it is unclear whether the results of this study may be extrapolated to pre-menopausal, at-risk women. In addition, the means whereby circulating IGF-I may affect breast carcinogenesis is still unclear. Recent findings suggest that the autocrine/paracrine activity of the growth factor is independent of its serum levels (Yakar et al, 1999). On the other hand, exogenous administration of IGF-I positively affects breast cell proliferation (Ng et al, 1997), supporting the hypothesis that circulating growth factor exerts a direct role on breast cell growth.

Given the putative role of IGF-I in breast carcinogenesis, the reduction of circulating IGF-I may be involved in the preventive effect of raloxifene. Debate is still open as to whether SERMs are true preventative agents or whether they treat the occult disease. Convincing arguments have been proposed in support of either mechanism, which may concur in reducing the clinical expression of the tumour (Radmacher and Simon, 2000). Consistent with this hypothesis, the early reduction of breast cancer incidence in the MORE trial may represent the suppression of subclinical cancer (Cummings et al, 1999), while the increasing separation of the curves of breast cancer occurrence up to 44 months (Cauley et al, 2001), suggests that the drug also exerts an early inhibitory effect on carcinogenesis, thus preventing the occurrence of a fully transformed phenotype (Brown and Lippman, 2000). Whatever the mechanism of raloxifene activity, a decrease of circulating IGF-I levels is desirable. In fact, although IGF-I has not been shown to predict postmenopausal breast cancer risk, the growth factor may intervene in different stages of breast carcinogenesis, since it induces proliferation, inhibits apoptosis and stimulates angiogenesis, thus favouring not only the appearance but also the maintainance of a transformed phenotype (Baserga, 1995; Yu and Rohan, 2000). Indeed, the evidence that the preventive activity of SERMs is restricted to ER-positive tumours and the reciprocal interaction between the oestrogen and the IGF-I signalling pathways suggest that the decrease of IGF-I may have some relevance, if any, in preventing the development of ER-positive breast cancer. However, the occurrence of a ER-negative phenotype is still unclear and the timing of ER changes in breast carcinogenesis appears to be very complex (Brown and Lippman, 2000).

In a similar manner to the results of the MORE trial, no dose–response effect was observed on IGF-I decline, suggesting that saturation of the oestrogen receptor occurs with the lower dose. The decrease in both IGF-I and proliferation, as assessed by Ki67 (Dowsett et al, 2001) may be possible mechanisms for the breast cancer preventive effect of raloxifene (Dowsett et al, 2001).

While the decline of circulating IGF-I may be desirable in breast cancer prevention, the implication of this finding for other situations is less clear. In addition to cancer, the IGF system is largely involved in bone formation and turnover. The circulating IGF system predicts the levels of markers of bone formation such as osteocalcin, although its relationship with bone mineral density (BMD) is less definite (Boonen et al, 1999; Collins et al, 1998). While lower levels of circulating IGF-I have been associated with an increased risk of vertebral fractures in postmenopausal women (Garnero et al, 1999), raloxifene administration for 3 years was associated with a 50% reduction in the risk of incident vertebral fractures in postmenopausal women despite the decrease in serum concentrations of osteocalcin (Ettinger et al, 1999) and in circulating IGF-I levels observed in the present study. Since mechanisms other than the increase of BMD may contribute to the anti-osteoporotic activity of raloxifene (Cummings, 2000) the decline of plasma IGF-I may not be a determining factor in this effect of the drug.

 *© 2001 Cancer Research Campaign*

In conclusion, raloxifene decreases circulating IGF-I and the IGF-I/IGFBP-3 ratio after short-term administration in postmenopausal women. The implication of these findings for the possible breast cancer preventive activity of raloxifene merits further investigation.

### ACKNOWLEDGEMENTS

The multicentric study was supported by Eli Lilly (Indianapolis, IN, USA). The Division of Chemoprevention is partially supported by the Italian Foundation for Cancer Research (FIRC). The authors are indebted to Ms Barbara Rossetti for patient management and to Mr William Russell-Edu for revising the English.

### REFERENCES

Baserga R (1995) The insulin-like growth factor-I receptor: a key to tumor growth? *Cancer Res* **55**: 249–252

Boonen S, Mohan S, Dequeker J, Aerssens J, Vanderschueren D, Verbeke G, Broos P, Bouillon R and Baylink DJ (1999) Down-regulation of the serum stimulatory components of the insulin-like growth factor (IGF) system (IGF-I, IGF-II, IGF binding protein [BP]-3, and IGFBP-5) in age-related (type II) femoral neck osteoporosis. *J Bone Miner Res* **14**: 2150–2158

Brown PH and Lippman SM (2000) Chemoprevention of breast cancer. *Breast Cancer Res Treat* **62**: 1–17

Burroughs KD, Dunn SE, Barrett JC and Taylor JA (1999) Insulin-like growth factor-I: a key regulator of human cancer risk (Editorial). *J Natl Cancer Inst* **91**: 579–581

Buzdar AU, Marcus C, Holmes F, Hug V and Hortobagyi G (1988) Phase II evaluation of Ly156758 in metastatic breast cancer. *Oncology* **45**: 344–345

Cauley JA, Norton L, Lippman ME, Eckert S, Krueger KA, Purdie DW, Farrerons J, Karasik A, Mellstrom D, Ng KW, Stepan JJ, Powles TJ, Morrow M, Costa A, Silfen SL, Walls EL, Scmitt H, Muchmore DB and Jordan VC (2001) Continued breast cancer risk reduction in postmenopausal women treated with raloxifene. 4-year results from the MORE trial. *Breast Cancer Res Treat* **65**: 125–134

Collins D, Woods A, Herd R, Blake G, Fogelman I, Wheeler M and Swaminathan R (1998) Insulin-like growth factor-I and bone mineral density. *Bone* **23**: 13–16

Cummings SR, Eckert S, Krueger KA, Grady D, Powles TJ, Cauley JA, Norton L, Nickelsen T, Bjarnason NH, Morrow M, Lippman ME, Black D, Glusman JE, Costa A and Jordan VC (1999) The effect of raloxifene on risk of breast cancer in postmenopausal women. *JAMA* **281**: 2189–2197

Cummings S (2000) The paradox of small changes in bone density vs large reduction in fracture risk. NIH Workshop on SERMs, National Cancer Institute 26–28 April, 2000, pp 126–127

Daughaday WH, Mariz IK and Blethen SL (1980) Inhibition of access of bound somatomedin to membrane receptor and immunobinding sites: a comparison of radioreceptor and radioimmunoassay of somatomedin in native and acid-ethanol – extracted serum. *J Clin Endocrinol Metab* **51**: 781–788

Decensi A, Bonanni B, Guerrieri-Gonzaga A, Gandini S, Robertson C, Johansson H, Travaglini R, Sandri MT, Tessadrelli A, Farante G, Salinaro F, Bettega D, Barreca A, Boyle P, Costa A and Veronesi U (1998) Biologic activity of tamoxifen at low doses in healthy women. *J Natl Cancer Inst* **90**: 1461–1467

Dowsett M, Bundred NJ, Decensi A, Sainsbury RC, Lu Y, Hills M, Cohen FJ, Veronesi P, O'Brien MER, Scott T and Muchmore DB (2001). Effect of raloxifene on breast cancer cell Ki67 and apoptosis: a double-blind, placebo-controlled, randomized clinical trial in postmenopausal patients. *Cancer Epidemiol Biomarkers Prev* **10**: 361–366

Ettinger B, Black DM, Mitlak BH, Knickerbocker RK, Nickelsen T, Genant HK, Christiansen C, Delmas PD, Zanchetta JR, Stakkestad J, Gluer CC, Krueger K, Cohen FJ, Eckert S, Ensrud KE, Avioli LV, Lips P and Cummings SR (1999) Reduction of vertebral fracture risk in postmenopausal women with osteoporosis treated with raloxifene. *JAMA* **282**: 637–645

Fisher B, Costantino JP, Wickerham DL, Redmond CK, Kavanah M, Cronin WM, Vogel V, Robidoux A, Dimitrov N, Atkins J, Daly M, Wieand S, Tan-Chiu E, Ford L, Wolmark N and other NSABP investigators (1998) Tamoxifen for prevention of breast cancer: report from the National Surgical Adjuvant Breast and Bowel Project P-1 Study. *J Natl Cancer Inst* **90**: 1371–1388

Garnero P, Sornay-Rendu E and Delmas PD (1999) Low serum IGF-I and occurrence of osteoporotic fractures in postmenopausal women. *Lancet* **355**: 898–899

Goldstein H (1995) Multivariate statistical models. Halsted Press: New York

Gradishar W, Glusman J, Lu Y, Vogel C, Cohen FJ and Sledge GW Jr. (2000) Effects of high dose raloxifene in selected patients with advanced breast carcinoma. *Cancer* **88**: 2047–2053

Hankinson SE, Willett WC, Colditz GA, Hunter DJ, Michaud DS, Deroo B, Rosner B, Spelzer FE and Pollak M (1998) Circulating concentrations of insulin-like growth factor-I and risk of breast cancer. *Lancet* **351**: 1393–1396

Helle SI, Anker GB, Tally M, Hall K and Lonning PE (1996) Influence of droloxifene on plasma levels of insulin-like growth factor (IGF)-I, Pro-IGF-IIE, insulin-like growth factor binding protein (IGFBP)-1 and IGFBP-3 in breast cancer patients. *J Steroid Biochem Mol Biol* **57**: 167–171

Kelloff GJ, Boone CW, Crowell JA, Steele VE, Lubet R and Doody LA (1994) Surrogate endpoint biomarkers for phase II cancer chemoprevention trials. *J Cell Biochem* **S9**: 1–9

Lonning PE, Hall K, Aakvaag A and Kien EA (1992) Influence of tamoxifen on plasma levels of insulin-like growth factor-I and insulin-like growth factor binding protein-1 in breast cancer patients. *Cancer Res* **52**: 4719–4723

Ng ST, Zhou J, Adesanya O, Wang J, LeRoith D and Bondy CA (1997) Growth hormone treatment induces mammary gland hyperplasia in aging primates. *Nat Med* **3**: 1141–1144

Pollak MN (1998) Endocrine effects of IGF-I on normal and transformed breast epithelial cells: potential relevance to strategies for breast cancer treatment and prevention. *Breast Cancer Res Treat* **47**: 209–217

Pollak M (2000) Insulin-like growth factor physiology and cancer risk. *Eur J Cancer* **36**: 1224–1228

Radmacher MD and Simon R (2000) Estimation of tamoxifen's efficacy for preventing the formation and growth of breast tumors. *J Natl Cancer Inst* **92**: 48–53

Toniolo P, Bruning PF, Akhmedkhanov A, Bonfrer JMG, Koenig KL, Lukanova A, Shore RE and Zeleniuch-Jacquotte A (2000) Serum insulin-like growth factor-I and breast cancer. *Int J Cancer* **88**: 828–832

Torrisi R, Pensa F, Orengo MA, Catsafados E, Ponzani P, Boccardo F, Costa A and Decensi A (1993) The synthetic retinoid fenretinide lowers plasma insulin-like growth factor I in breast cancer patients. *Cancer Res* **53**: 4769–4771

Vadgama JV, Wu Y, Datta G, Khan H and Chillar R (1999) Plasma insulin-like growth factor-I and serum IGF-binding protein 3 can be associated with the progression of breast cancer and predict the risk of recurrence and the probability of survival in African–American and Hispanic women. *Oncology* **57**: 330–340

Veronesi U, De Palo G, Marubini E, Costa A, Formelli F, Mariani L, Decensi A, Camerini T, Rosselli Del Turco M, Di Mauro MG, Muraca MG, Del Vecchio M, Pinto C, D'Aiuto G, Boni C, Campa T, Magni A, Miceli R, Perloff M, Malone WF and Sporn MB (1999) Randomized trial of fenretinide to prevent second breast malignancy in women with early breast cancer. *J Natl Cancer Inst* **91**: 1847–1856

Yakar S, Liu JL, Stannard B, Butler A, Accili D, Sauer B and LeRoith D (1999) Normal growth and development in the absence of hepatic insulin-like growth factor I. *Proc Natl Acad Sci USA* **96**: 7324–7329

Yu H and Rohan T (2000) Role of the insulin-like growth factor family in cancer development and progression. *J Natl Cancer Inst* **92**: 1472–1489

© 2001 Cancer Research Campaign
